# Experimental Investigation on Laser Assisted Diamond Turning of Binderless Tungsten Carbide by In-Process Heating

**DOI:** 10.3390/mi11121104

**Published:** 2020-12-14

**Authors:** Kaiyuan You, Fengzhou Fang, Guangpeng Yan, Yue Zhang

**Affiliations:** 1 Centre of Micro/Nano Manufacturing Technology (MNMT), State Key Laboratory of Precision Measuring Technology & Instruments, Tianjin University, Tianjin 300072, China; youkaiyuan@tju.edu.cn (K.Y.); gpyan@tju.edu.cn (G.Y.); yuede1993@163.com (Y.Z.); 2 Centre of Micro/Nano Manufacturing Technology (MNMT-Dublin), School of Mechanical & Materials Engineering, University College Dublin, Dublin 4, Ireland

**Keywords:** laser assisted turning, tungsten carbide, diamond turning, finite element analysis

## Abstract

Binderless tungsten carbide (WC) finds widespread applications in precision glass molding (PGM). Grinding and polishing are the main processes to realize optical surface finish on binderless WC mold inserts. The laser assisted turning (LAT) by in-process heating is an efficient method to enhance the machinability of hard and brittle materials. In this paper, laser heating temperature was pre-calculated by the finite element analysis, and was utilized to facilitate laser power selection. The effects of rake angle, depth of cut, feed rate, and laser power are studied experimentally using the Taguchi method. The variance, range, and signal-to-noise ratio analysis methods are employed to evaluate the effects of the factors on the surface roughness. Based on the self-developed LAT system, binderless WC mold inserts with mirror finished surfaces are machined using the optimal parameters. PGM experiments of molding glass lenses for practical application are conducted to verify the machined mold inserts quality. The experiment results indicate that both the mold inserts and molded lenses with the required quality are achieved.

## 1. Introduction

The binderless tungsten carbide (WC), as the typical hard and brittle material, has been widely applied in the field of optics, photonics and life science owing to its ideal physical properties [1]. However, the ultra-high hardness and brittleness of the WC material also decrease the machinability and greatly limit the feasible machining approaches. At present, mirror finished surfaces of binderless WC mainly relying on grinding and polishing, which is time consuming and infeasible for large-sag concave surface shape [2]. There is a bottleneck of severe tool wear in using single point diamond turning (SPDT) [3]. Too small of a critical ductile-to-brittle transformation (DBT) thickness of hard and brittle materials [4] also brings high cost and labile turning surface integrity [5]. Thus, the more reliable and efficient approach of machining binderless WC is urgent [6,7].

On account of the materials’ improved machinability at elevated temperature, laser assisted turning (LAT) is reliable for hard and brittle material machining and is getting more applications. With this approach, the machining efficiency, cutting force, and tool wear can be greatly improved. At present, there are two primary kinds of laser assisted turning forms, including pre-heat laser assisted turning (Pre-LAT) and in-process-heat laser assisted turning (In-LAT) [8]. The Pre-LAT method locates the laser spot above the cutter and pre-heat the cutting region material ahead of tool interaction. Pre-LAT has a mature development and is widely applied in the traditional cylindrical turning [9] but restricted in the field of end-face ultra-precision diamond turning due to the structural constraint. On the other hand, the In-LAT method guides the laser beam passes through the transparent tool to heat material locally, thereby decreasing the hardness and altering the fracture toughness as soon as the tool interacts with the material [10]. The In-LAT technology brings better machinability, makes the ductile turning of hard and brittle materials possible, and has been a consummate choice for hard and brittle material ultra-precision machining.

In the past decade, there have been an increasing number of studies carried out on the In-LAT of hard and brittle materials. Langan et al. [11] study the laser parameter effects of In-LAT machining on residual stress and phase purity of monocrystalline silicon. Chen et al. [12] also analyze monocrystalline silicon subsurface damage and phase transformation caused by In-LAT through tapper cutting experiment and molecular dynamic simulation. Langan et al. [13] prove the in-process laser heating can reduce the residual stress of sapphire surface finishing and further demonstrating the applicability of the In-LAT method on nominally transparent brittle materials. Park et al. [14] discuss the surface finish and cutting force improvement of bulk metallic glass using sapphire tool with the assistance of laser in-process heat and textured rake face. Di et al. [15] performed In-LAT experiments, proving the In-LAT method can promote the ductile mode material removal of WC, but without lucubrated analysis and discussion. So far, little study discusses the In-LAT parameter effects on the surface quality of binderless WC.

Finite element analysis (FEA) is a superior method to calculate the thermal field based on the numerical calculation [16]. Since the measurement resolution of commercial thermal imaging cameras is restricted by the diffraction limit, the peak temperature during laser heating and nanoscale machining process cannot be measured precisely, but can be calculated using the FEA method. Kamlesh et al. [16] calculate the silicon temperature distribution under Gaussian profile laser beam heating regarding the material high-pressure phase change. Li et al. [17] compare the temperature field of Al_2_O_3_ ceramic material with volumetric and surface laser heating sources. Shang et al. [18] utilize the three-dimensional transient heat conduction model to predict the temperature distribution caused by a freeform laser trajectory, whose results inversely facilitate the laser parameter selections in the laser assisted milling. At present, there is no study has discussed the temperature field of binderless WC under spiral moving laser heating, which can guide the In-LAT laser parameters selection.

Various parameters of In-LAT have a significant influence on the binderless WC mold inserts finish quality, but to our best knowledge, there is little comprehensive discussion until now. In this study, the laser power heating temperature was analyzed using both the numerical calculation and FEA, acquiring the approximate range of optimal laser power. Furthermore, the experiment design methodology, the Taguchi design, was utilized to explore the optimal In-LAT parameters for binderless WC. The effects of tool rake angle, machining depth of cut (DoC), feed rate, and laser power were studied experimentally. The analysis of variance (ANOVA), signal to noise (S/N) ratio, and range analysis methods were utilized to select the optimal parametric combination and verify the validity of the experiment. With the optimal parameters, binderless WC mold inserts were machined based on the self-developed LAT system, and were used in the precision glass molding (PGM) experiment subsequently. Both the mold inserts and molded lenses with ideal surface quality were achieved, verifying the In-LAT machined mold insert can be successfully applied for the replicative mass production of glass lenses with mirror finished surface.

The rest of this paper is organized as follows. Section 2 calculates the workpiece temperature under various laser power heating. Section 3 introduces the experimental setup and the parameter design. The analysis of the Taguchi experiment result is described in Section 4.1. The final mold inserts machined by the In-LAT method and the PGM experiment are presented in Section 4.2. Finally, the major conclusions and the following works are presented in Section 5.

## 2. Thermal Field Analysis of Laser Heating

The material’s temperature is a vital index for In-LAT and has a considerable impact on the machining quality. The appropriate heating temperature could decrease the material’s hardness and increase the machinability, but the excess temperature will introduce undesired thermal damage and severe diamond tool wear. It is essential to maintain the laser heating temperature within the optimal range, which is mainly dependent on the irradiated laser power. The numerical calculation could facilitate the thermal field analysis and economize the experimental works. Considering the thermal initial condition and complex boundary conditions, the workpiece thermal field can be precisely calculated. Before the laser heating, the initial thermal condition of the WC workpiece can be determined by the uniform temperature *T*_0_ as

(1) $$T(0,t)=T_{0}.$$

There are many boundary conditions during the laser heating process, which can be divided into the laser heating effect, thermal radiation [19], and cutting fluid convection [20]. For the Gauss laser spot on the *z* = 0 plane, the heat flux of spiral trajectory moving laser spot can be described as

(2) $$q_{l}(x,y,0,t)=\frac{2P\delta }{\pi r^{2}}\exp\left\{\frac{2\left\{{\left[x-\left(x_{0}-ft\right)\cos\left(\pi nt/30\right)\right]}^{2}+{\left[y-\left(x_{0}-ft\right)\sin\left(\pi nt/30\right)\right]}^{2}\right\}}{r^{2}}\right\},$$

where *P* (W) refers to the laser power, *r* (μm) is the laser spot radius, *δ* is the laser absorptivity, and the laser scanning path can be determined by the initial position (*x*_0_,0), feed rate *f* (μm/rev), and rotation speed *n* (rev/min). In addition, the cutting fluid convection can be calculated by

(3) $$q_{c}\left(x,y,z,t\right)=h(T_{f}-T),$$

where *h* (W·m^−2^·K^−1^) represents the convective heat transfer coefficient and should be measured by the experiment, *T_f_* refers to the cutting fluid temperature. Thermal radiation is

(4) $$q_{r}\left(x,y,z,t\right)=\sigma \varepsilon (T_{amb}^{4}-T^{4}),$$

where the *σ* = 5.67 × 10^−8^ W/(m^2^·K^4^) is the Stefan-Boltzmann constant, and the *ε* represents the surface emissivity, *T_amb_* means the ambient temperature. The basic equation for the analysis of heat conduction is the Fourier’s law

(5) $$q_{n}=-K_{n}\frac{\partial T}{\partial n},$$

where heat flux *q_n_* is the heat transfer rate in the *n* direction per unit area perpendicular to the direction of heat flow. *∂T/∂n* (K/m) is the temperature gradient in the direction *n*. The *K_n_* (W·m^−1^·K^−1^) is the thermal conductivity in *n* direction. However, the workpiece material is thought to be isotropic in this study, indicating that the *K_n_* is a constant value. Owing to there being no volumetric energy addition, so the three-dimensional thermal conduction within the workpiece can be calculated by

(6) $$\frac{\partial T}{\partial t}=k\nabla^{2}T,$$

where *k* is the thermal diffusion coefficient in m^2^/s, satisfying *k* = *K*/(*ρ*·*C_p_*). *K* refers to the material thermal conductivity coefficient, *ρ* represents the density of the material (kg/m^3^), and *C_p_* is the specific heat (J/kg·K) of the material.

**Figure 1:**
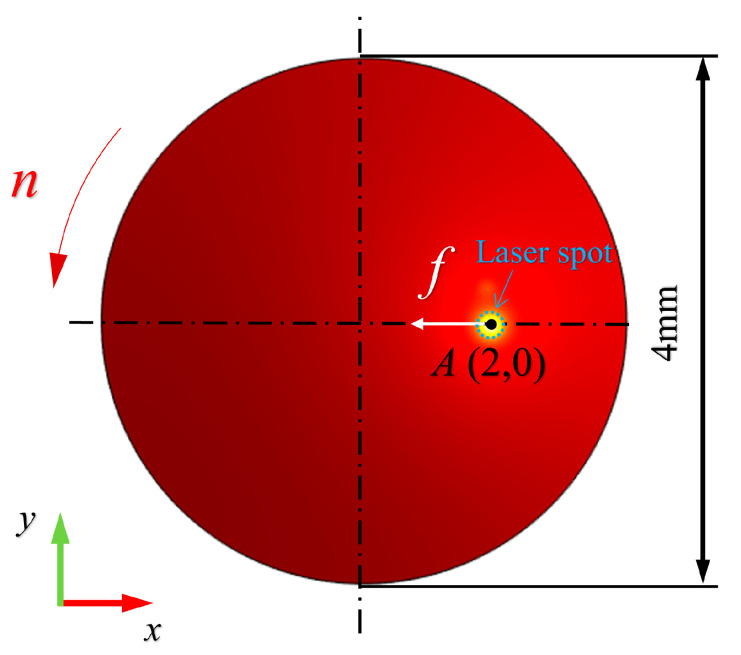
Finite element analysis (FEA) model of laser heating.

FEA is a powerful numerical calculation strategy, and was utilized to compute the temperature field as shown in Figure 1. The laser heating WC workpiece model was established with the assistance of COMSOL software. The triangular mesh type was adopted and firstly generated in the workpiece upper surface and then sweep in the thickness direction. In particular, the FEA mesh characteristic size was controlled within 1 μm to 40 μm to avoid the tedious calculation time and severe calculation distortion. The workpiece coordinate system was established with the origin coincident with the workpiece center. The WC workpiece is regarded as isotropic with 4 mm diameter and 0.5 mm thickness. Furthermore, the 1064 nm continuous wave laser with a radius of 85 μm is loaded on the workpiece upper surface (*z* = 0). The laser energy distribution conforms to the Gauss theorem. The relative motion between laser spot and workpiece in the FEA model is consistent with the machining scene. The laser spot feeds along *-x* direction with 1 mm/min speed while the workpiece rotates with the constant 2000 rev/min. The typical thermodynamic parameters such as laser absorptivity refer to the pre-researchers’ work, which ensures the accuracy and reliability of the simulation results. The initial workpiece temperature is assumed to be 293 K. For clarity, the FEA simulation parameters have been summarized in Table 1.

**Figure 2:**
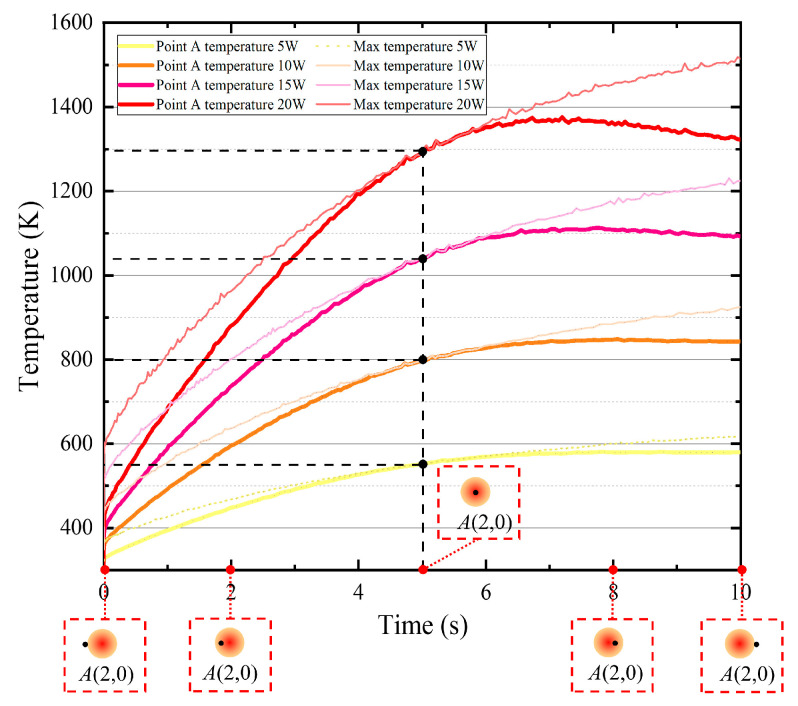
Historical temperature information of binderless tungsten carbide (WC) workpiece under different laser power heating.

Since the laser spot center is coincident with the diamond tool tip and the radius of laser spot (85 μm) is much larger than the feed distance per cycle (0.5 μm), the laser heat accumulation effect should be considered. The laser heating process should be computed when the laser spot covers the specific machining point. For the specifical point *A*(2,0), the workpiece was heated by the moving laser beam from the initial position (2.085,0) of beam center to the end position (1.915,0) with the 1mm/min velocity. The FEA model calculates the thermal field of the laser heating with four power levels (5 W, 10 W, 15 W, 20 W) based on Equations (1)–(6). The workpiece highest temperature, and the temperature history of specific point *A* have been recorded and plotted in Figure 2. When the laser beam center moves to the *A* point, the temperature of point *A* is same as the highest temperature, which increase to 554.0 K, 802.2 K, 1044.7 K, and 1294.9 K for 5 W, 10 W, 15 W, and 20 W laser power respectively. If the workpiece temperature is much higher than the diamond graphitization temperature 1000 K [22], it will result in severe tool wear and thermal damage. Thus, higher laser power will not be considered. The parameters of 5 W, 10 W, 15 W, 20 W were adopted in the following orthogonal experiment.

## 3. Experimental Approach

### 3.1. In-Process-Heat Laser Assisted Turning (In-LAT) Experiment Setup

**Figure 3:**
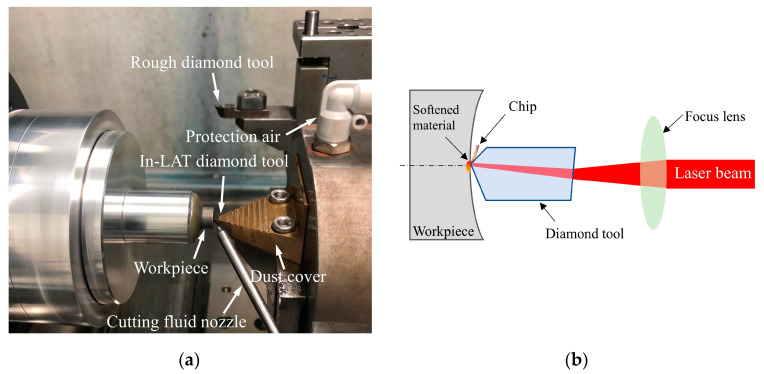
(**a**) The experimental setup, and (**b**) the schematic diagram of the in-process-heat laser assisted turning (In-LAT) system.

The experiment was carried on based on an ultra-precision three-axis lathe with the self-developed LAT system as shown in Figure 3a. The *x*-axis and *z*-axis of lathe drive the spindle and diamond tool moves linearly in *x* and *z* directions, respectively. The binderless WC was mounted on the aluminum substrate and vacuum-chucked onto the lathe spindle. The workpiece has a concave spherical surface with 7.5 mm diameter and 7 mm curvature, and rotates with the lathe spindle, realizing the cutting movement and material removal.

The self-developed LAT system was set up on the *z*-guide platform of lathe without deflection. There are several primary parts of the LAT system, including the laser source, optical elements, positioning device, tool holder, and In-LAT diamond tool. Specifically, the Nd: YAG fiber laser can generate continuous wave laser with 1064 nm wavelength, which possesses the Gaussian power distribution (M^2^ = 1.126) and can adjust within the range of 2–100 W. The emitted laser is guided into the optical system through the laser fiber, thereby focusing the laser beam into the minimum 14 μm diameter laser spot and can also be enlarged by defocusing. The multi-axis positioning device can drive the optical system to move along three vertical directions realizing the laser spot position alignment. For the purpose of protecting optical lenses, the cutting chips are separated from the optical system via the protection air and dust cover. Moreover, since the natural diamond has an inherent variability in mechanical and optical properties, the chemical vapor deposition (CVD) monocrystalline diamond with extremely uniform properties was chosen to fabricate the In-LAT diamond tool.

### 3.2. Parameters Design

Taguchi method is an experimental design methodology to study the effects of multi-factors and multi-levels and was used for investigating the In-LAT optimal laser power and machining parameters of binderless WC in this study. The experimental parameters were designed referring to orthogonal array, whose factors are independent of each other and can be evaluated separately. The effect of one individual factor would not affect the estimation of other factors. Taguchi method can be employed to greatly reduce experiments by selecting some representative conditions from the comprehensive experiment according to the orthogonality.

There are various factors that have a significant influence on the surface finish quality, including the machining parameters and laser parameters. The effects of laser power, tool rake angle, DoC, and feed rate are considered with 5 levels herein as listed in Table 2. The level values of machining factors are determined by the preliminary experiments. The laser powers are selected by the numerical calculation results. In particular, the blank group was designed for the variance analysis and separating the impact of the accidental error. Finally, the L_25_(5^5^) standard orthogonal array was selected and utilized to perform experiments.

**Figure 4:**
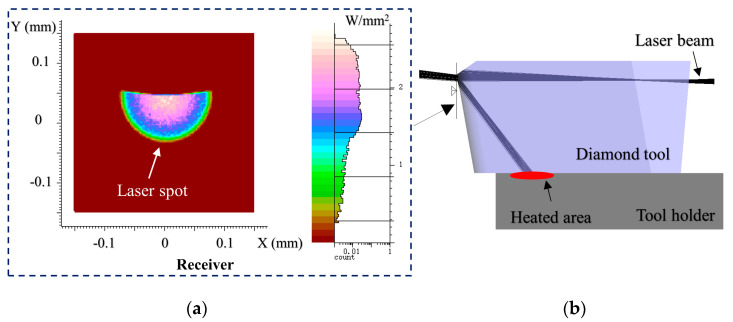
Optical simulation results. (**a**) Emitted laser spot from diamond tool and (**b**) laser beam path in the In-LAT diamond tool.

In order to ensure the rationality of the orthogonal experiment, the rest machining parameters remain constant as listed in Table 3. Experiments were conducted on the same workpiece, which rotates with a spindle speed of 2000 rpm. The diamond tools with a nominal 10° clearance angle and 0.3 mm nose radius were used. Each orthogonal experiment ensured that the cutting edges of the In-LAT diamond tools are in good condition and have the homogeneous quality. Moreover, an 85 μm radius laser spot was employed for enough laser beam cover angle on the cutting edge, and was obtained with the 1.96 mm defocusing length. The laser spot position has been accurately adjusted where the laser spot center is coincident with the diamond tool tip. For facilitating the visual analysis, the laser beam path at the ideal position was modeled by the optical simulation. Almost the half laser beam is reflected on the tool rake face owing to the total reflection principle, the emitted laser spot pattern is plotted in Figure 4a with semi-Gauss energy distribution. The reflection part of laser beam continuously heats the tool holder as shown in Figure 4b. All the experiments were carried on using the same cutting fluid (ISOPAR-H) assistance in the same fluid spraying direction. The deep thermal damage layer caused by high-power laser was pre-removed by the rough diamond tool. The workpiece surfaces before turning were polished using the diamond paste (grain size 0.5 μm) before each orthogonal experiment, guaranteeing the consistency of workpiece surface quality.

## 4. Results and Discussion

### 4.1. Effect of Test Parameters on Surface Roughness

In this study, the surface roughness was chosen as the evaluation index of surface finish quality. The surface roughness of the machined workpiece was measured by a laser confocal microscope (OLYMPUS LEXT OLS 4000, Olympus, Tokyo, Japan) with 20× objective lenses and 4× digital zoom. The scanning field is set as (161 × 161 μm^2^). All measurement data have been disposed with the 80 μm high-pass filter to remove the intermediate frequency signal introduced by the surface form. Surfaces machined by each parameter group were measured three times to eliminate the effect of the accidental error. The mean roughness has been utilized to analyze the orthogonal experiment factors’ impact on the surface finish quality.

#### 4.1.1. Analysis of Variance

The ANOVA method can be used to distinguish the difference between the test results caused by the factor variation from the impact of the accident error, and can give a reliable quantitative estimate. Based on the measurement results, ANOVA was applied to study the significance of the input factors on the surface roughness at a 95% confidence level. The factors’ degree-of-freedom, sum-of-squares, mean-of-squares, *F*-value, *p*-value, and contribution rate are calculated and listed in Table 4.

The *p*-value reflects the significance of orthogonal factors. The factor with a smaller *p*-value indicates it has a more significant influence on the experimental results. The *p*-values of rake angle, DoC, feed rate, and laser power are 0.021, 0.402, 0.514, and 0.269 respectively. In particular, the ANOVA results declare that the blank group has a larger influence than the DoC and feed rate, indicating their effect is not significant within the designed parameter range. The contribution rate of rake angle and laser power counts 48.85% and 14.42%, respectively. It is evident that the rake angle and laser power are the main factors affecting the surface roughness during the binderless WC machining, and that the rake angel of diamond tool is the most influential factor.

#### 4.1.2. Analysis of the Signal-to-Noise Ratio and Mean Value

**Figure 5:**
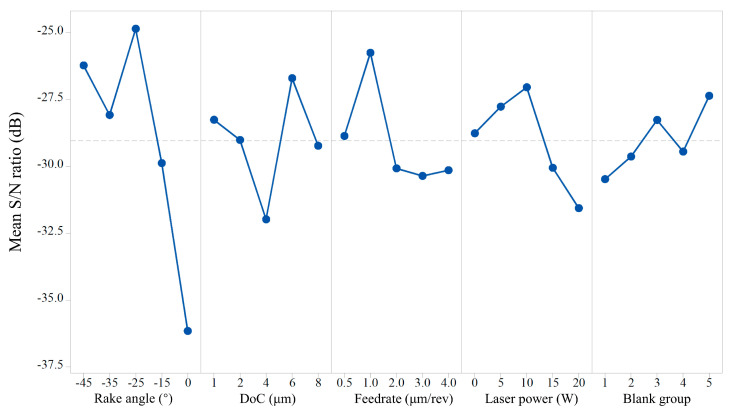
Main effect of mean S/N ratios corresponding to the surface roughness.

The S/N ratio is an important index of the Taguchi design robustness. A reasonable parameter combination with a minimize noise factor effect can be selected through the results analysis, thereby ensuring the stability of the machining process. If the value of the signal-to-noise ratio (S/N) is larger, the effect of the experimental noise factor under this parameter value is smaller. In this study, the surface roughness is chosen as the evaluated index and desired to be small. Thus, the principle of the smaller-the-better was adopted in the S/N ratio analysis. The effects of individual factors on the surface roughness characteristics were analyzed as listed in Table 5. The analysis results indicate that the parameter combination of −25° rake angle, 6 μm DoC, 1 μm/rev feed rate, and 10 W laser has minimal effect of noise factor as shown in Figure 5. The delta of the blank group presents the smallest value (3.12 dB), verifying the validity of the experiment design.

**Figure 6:**
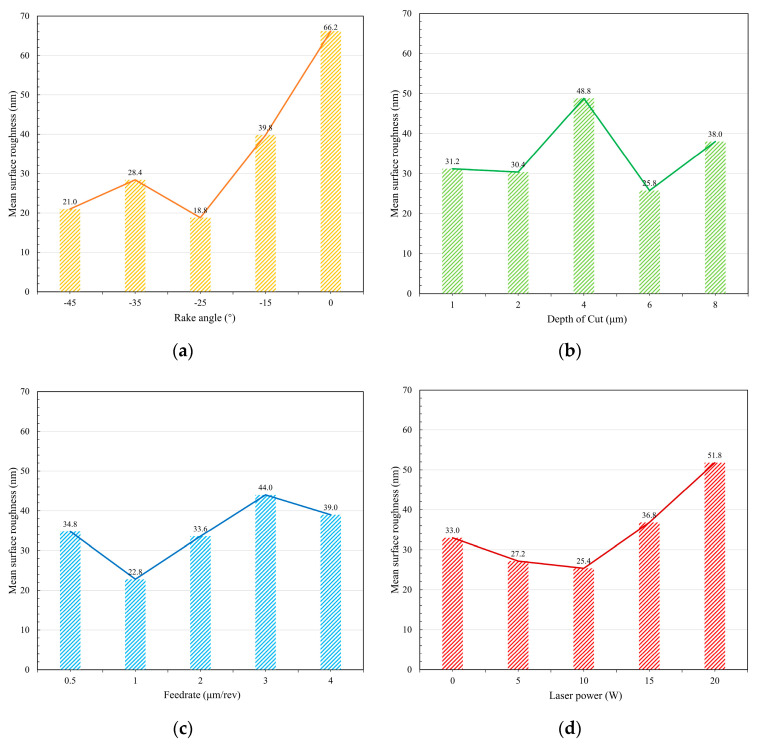
Surface roughness correlation with the variation of (**a**) rake angle, (**b**) DoC, (**c**) feed rate, and (**d**) laser power.

**Figure 7:**
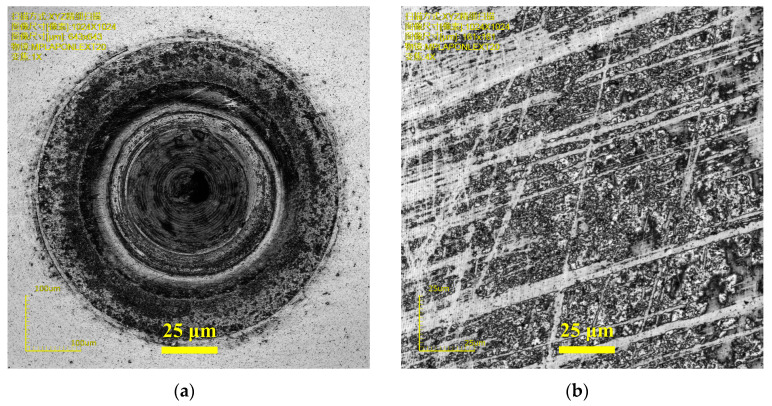
Laser microscope images of (**a**) thermal damage area under 20 W laser; (**b**) high-temp chip stuck on the machined surface.

In order to optimize the experimental parameters, the statistical results of factor impact have been analyzed. The mean value and delta of surface roughness for individual factors at the same level have been calculated as summarized in Table 5. In particular, the delta of the blank group has the biggest value, verifying the experiment validity again. The experimental results declare the −25° tool rake angle has the minimum surface roughness as shown in Figure 6a. The negative rake angle introduces large hydrostatic stress, which is beneficial for the high-pressure phase transformation of hard and brittle materials [23] and has much better results than zero-rake angle tools. However, the large negative rank angle tool can also bring the difficulty of chip removal and large cutting force which also result in the deteriorating machining quality. Besides, the 6 μm DoC has a lower surface roughness as shown in Figure 6b. However, the DoC effect of the experiment has obvious randomicity, indicating the DoC has a slight influence on the surface roughness within the given level range. On the other hand, 1.0 μm/rev is thought to be the optimal feed rate of binderless WC machining as shown in Figure 6c. The small feed rate makes the undeformed chip thickness (UCT) smaller than the critical DBT depth of WC. However, the smallest feed rate level (0.5 μm/rev) is so small that resulted in UCT is likely to smaller than the tool edge radius and brings a large effective negative rake angle [24]. Moreover, the optimal surface quality was obtained with 10 W laser power as shown in Figure 6d. There is obvious thermal damage and deteriorate machining quality under 20 W laser power as shown in Figure 7a. Furthermore, since the laser heating locally, there are some stuck high-temp chips on the machined WC surface. The stuck chip can be removed by the polishing process but not work for wipe as shown in Figure 7b. From this discussion, it can be concluded that the optimal parametric combination for minimum surface roughness was the group of the −25° tool rake angle, 6 μm DoC, 1.0 μm/rev feed rate, and 10 W laser power, which is consistent with the result of S/N ratio analyze.

### 4.2. Mold Insert and Molded Glass Quality

**Figure 8:**
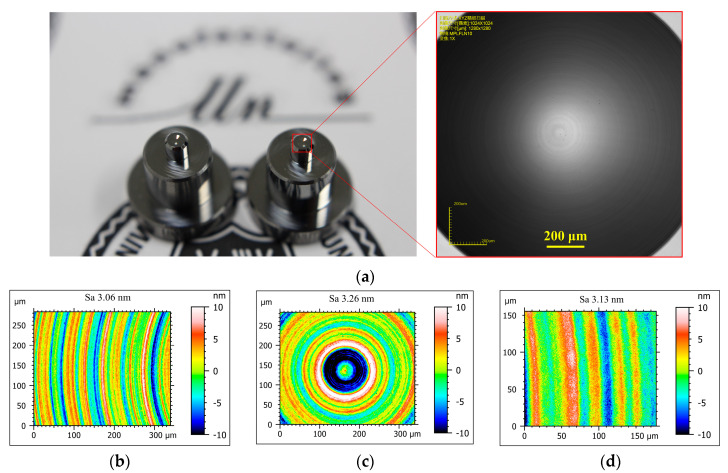
In-LAT machined mold inserts, (**a**) the measured mold surface morphology, (**b**) the fringe area, (**c**) central area, and (**d**) outer platform area.

**Figure 9:**
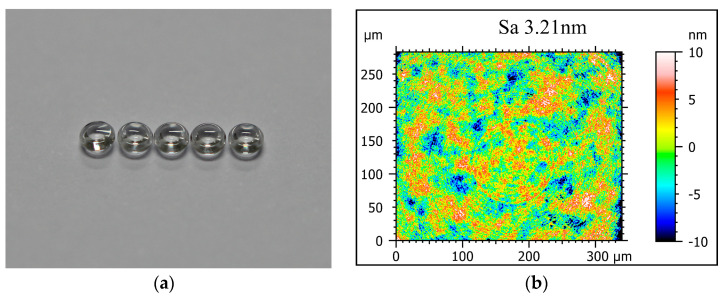
Molded lenses (**a**) the molded lenses, (**b**) the measured surface morphology of the central region.

With the obtained optimal parameters, two concave mold inserts with ideal surface quality have been machined on binderless WC via the self-developed LAT system as shown in Figure 8a. The inserts possess a 1.62 mm diameter concave aspherical surface and 0.4 mm width platform. Two inserts were machined using the same diamond tool seven times to verify the parametric stability. In order to verify the mold inserts’ quality, the PGM experiment was performed on an aspherical lens molding machine (DTK-LMR-3300 V2, Daehoteck, Changwon-si, Korea). The glass selected for the molding experiment was a moldable glass (D-ZLAF52LA, CDGM, Chengdu, China), whose transition temperature is 546 °C. Molded lenses were produced under 0.3 Mpa pressure and 586 °C temperature as shown in Figure 9a.

#### 4.2.1. Surface Finish

Both the mold inserts and molded lenses with homogeneous quality are achieved, and the surface morphology was measured by a white light interferometer (S Neox, Sensofar, Barcelona, Spain). The 20× objective lenses were used under the VSI measurement mode for the moderate scanning field (0.340 × 0.283 μm^2^). The measurement data has also been disposed with the 80 μm high-pass filter to remove the intermediate frequency signal introduced by the surface form. The edge part is clipped to suppress the edge effect of filtering. Furthermore, the fringe area of mold inserts possesses the surface roughness of *Sa* 3.06 nm as shown in Figure 8b. The center area presents larger surface roughness *Sa* 3.26 nm owing to the tool setting error and slow cutting speed as shown in Figure 8c. Moreover, the smaller field (0.176 × 0.154 μm^2^) has been measured with *Sa* 3.13 nm as shown in Figure 8d on the platform of mold insert owing to the narrow width of the platform. The molded glass optics have homogeneous quality with the mold insert as shown in Figure 9b, with the ideal surface quality of *Sa* 3.21 nm.

#### 4.2.2. Form Error

**Figure 10:**
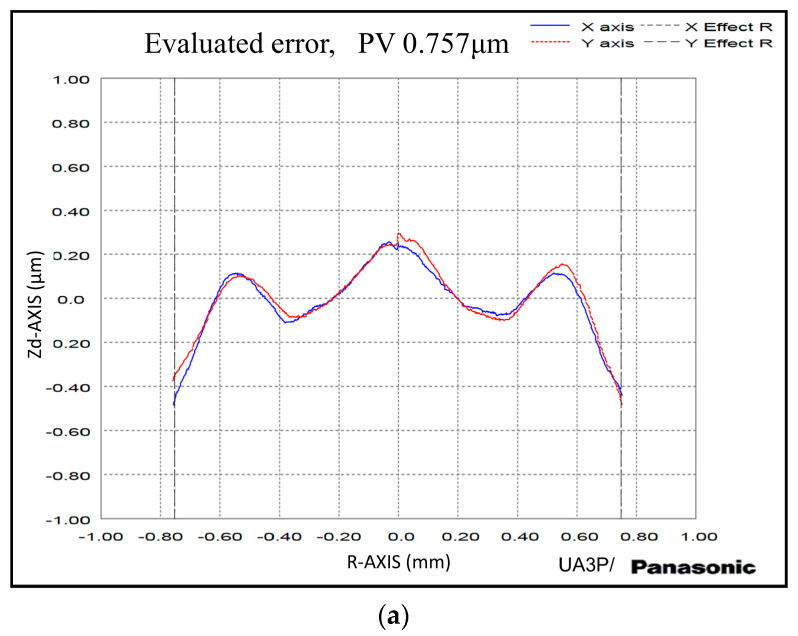

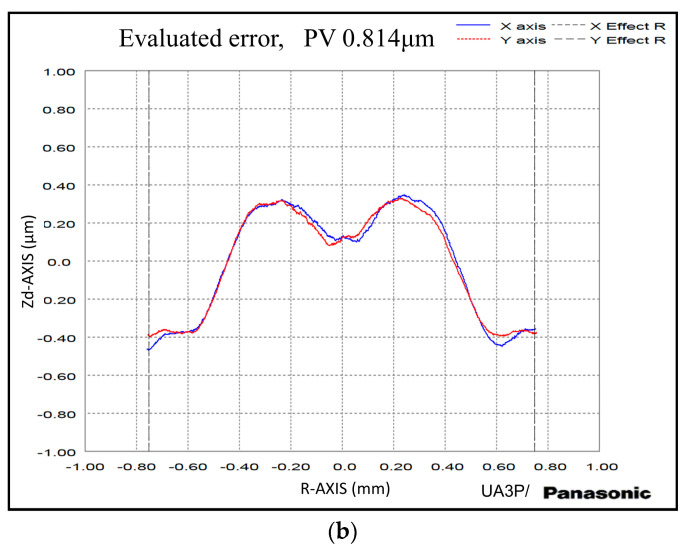
The form error of (**a**) the mold inserts and (**b**) molding lenses.

The form error of mold inserts and molded lenses were measured using a form measurement instrument (UA3P-300, Panasonic, Osaka, Japan). The diamond probe with a radius of 2 μm was used to scan the optics at a scanning speed of 0.2 mm/s. Since almost half part of the laser beam total reflect on the tool rake face and absorbed by the tool holder as shown in Figure 4, the tool holder is heated continuously during the In-LAT machining process, thereby introducing inevitable thermal expansion, and then resulted in poor alignment accuracy. The introduced tool setting error makes the form error of mold insert is not ideal with PV 0.757 μm as shown in Figure 10. The molded lenses also present a complementary form error pattern with PV 0.814 μm, which is unqualified for the practical application.

## 5. Conclusions

The laser power heating temperature was analyzed with the numerical calculation and FEA model firstly, acquiring the approximate range of optimal laser power. The orthogonal In-LAT experiment with the statistical analysis method of AVONA, S/N ratio, and range analysis was conducted to study the effect of tool rake angle, machining DoC, feed rate, and laser power. The optimal parametric combination for minimum surface roughness and maximal S/N ratio was achieved using the −25° tool rake angle, 6 μm DoC, 1.0 μm/rev feed rate, and 10 W laser power. With the optimal parameters, the mold inserts with ideal surface quality were machined on the binderless WC based on the self-developed In-LAT system. The PGM experiment was performed subsequently to verify the mold inserts’ quality. The experiment results indicate that surface roughness of 3.26 nm and 3.21 nm in Ra have been achieved on the central area of inserts and lenses respectively, verifying the superiority and robustness of the optimal parametric combination. The form error of insert and lenses with PV 0.757 μm and PV 0.814 μm were obtained respectively, which has room for further improvement in the following investigation.

## Figures and Tables

**Table 1:** Finite element analysis (FEA) simulation parameters.

Parameters	Value
Laser wavelength (*λ*)	1064 nm
Laser power (*P*)	5 W, 10 W, 15 W, 20 W
Laser spot radius (*r*)	85 μm
Laser translational velocity (*f*)	1 mm/min
Workpiece material	Tungsten carbide
Rotation speed (*n*)	2000 rev/min
Workpiece diameter	4 mm
Workpiece thickness	0.5 mm
Initial temperature (*T*_0_)	293 K
Emissivity (*ε*)	0.75 [19]
Absorptivity (*δ*)	0.23 [21]
Convective heat transfer coefficient (*h*)	300 W/(m^2^·K) [20]

**Table 2:** Orthogonal parameters and corresponding values

Factors	Units	Levels of Factors				
		Level 1	Level 2	Level 3	Level 4	Level 5
Rake angle	°	0	−15	−25	−35	−45
Depth of cut	μm	1	2	4	6	8
Feed rate	μm/rev	0.5	1	2	3	4
Laser power	W	0	5	10	15	20
Blank group		1	2	3	4	5

**Table 3:** Constant machining parameters

Parameters	Description
Workpiece material	Binderless tungsten carbide
Rotation speed	2000 rpm
Tool material	CVD monocrystalline diamond
Clearance angle	10°
Nose radius	0.3 mm
Laser spot radius	85 μm
Laser position	Beam center coincides with tool tip
Cutting fluid	ISOPAR-H

**Table 4:** Analysis of variance (ANOVA) results from mean surface roughness

Factors	Degree-of- Freedom	Sum-of- Squares	Mean-of- Squares	*F*-Value	*p*-Value	Contribution Rate
Rake angle	4	7491.8	1872.9	5.36	0.021	48.85%
DoC	4	1597.8	399.4	1.14	0.402	10.42%
Feed rate	4	1238.6	309.6	0.89	0.514	8.08%
Laser power	4	2211.8	552.9	1.58	0.269	14.42%
Error	8	2795.5	349.4	-	-	18.23%
Total	24	15335.4	-	-	-	-

**Table 5:** Response table for mean surface roughness

Control Factors	Resultant Surface Roughness						
	Level 1	Level 2	Level 3	Level 4	Level 5	Delta	Row Rank
Mean of S/N ratio (dB)							
Rake angle	−26.22	−28.08	−24.86	−29.88	−36.15	11.30	1
DoC	−28.26	−29.01	−31.98	−26.70	−29.23	5.28	2
Feed rate	−28.86	−25.75	−30.08	−30.36	−30.14	4.61	3
Laser power	−28.76	−27.77	−27.04	−30.05	−31.56	4.52	4
Blank group	−30.48	−29.63	−28.26	−29.45	−27.36	3.12	5
Mean of surface roughness (nm)							
Rake angle	21.00	28.40	18.80	39.80	66.20	47.40	1
DoC	31.20	30.40	48.80	25.80	38.00	23.00	3
Feed rate	34.80	22.80	33.60	44.00	39.00	21.20	4
Laser power	33.00	27.20	25.40	36.80	51.80	26.40	2
Blank group	46.4	33.20	27.80	32.80	34.00	18.60	5
